# Octacosanol: A Natural Bioactive Ingredient for Atherosclerosis Prevention and Cardiovascular Health Promotion

**DOI:** 10.3390/molecules31142451

**Published:** 2026-07-13

**Authors:** Xiuli Yang, Haixia Han, Zixuan He, Mingxi Jia

**Affiliations:** 1 College of Food Science and Pharmacy, Xinjiang Agricultural University, Urumqi 830052, China; 2 College of Modern Industry in Chinese Herbal Medicine, Xinjiang Agricultural University, Urumqi 830052, China

**Keywords:** octacosanol, atherosclerosis, antioxidant activity, lipid metabolism, endothelial function

## Abstract

Cardiovascular diseases remain the top cause of death worldwide, with atherosclerosis as a key underlying factor. Natural bioactive ingredients from functional foods are increasingly sought after for preventing chronic metabolic and heart conditions due to their safety and suitability for long-term use. Octacosanol is a naturally occurring long-chain fatty alcohol primarily isolated from plant waxes, including rice bran wax and sugarcane wax. Emerging evidence indicates that octacosanol exhibits promising antioxidant properties and may exert protective effects against atherosclerosis through modulation of lipid metabolism and endothelial function. Unlike statins, which can cause side effects over time, octacosanol works gently through several pathways: it protects blood vessel lining by clearing reactive oxygen species and activating endothelial nitric oxide synthase (eNOS); reduces vascular inflammation by blocking nuclear factor kappa-B (NF-κB) and mitogen-activated protein kinase (MAPK) signaling; moderates lipid metabolism by lowering 3-hydroxy-3-methylglutaryl-coenzyme A (HMG-CoA) reductase activity and proprotein convertase subtilisin/kexin type 9 (PCSK9) expression; and suppresses excessive platelet aggregation to reduce thrombotic risk. When taken alongside statins, it boosts lipid control while easing statin-related side effects. With its excellent safety profile, octacosanol is an ideal natural ingredient for functional foods, offering a novel multi-target dietary approach to support long-term cardiovascular health.

## 1. Introduction

Cardiovascular diseases (CVDs) remain the leading global public health burden, with their incidence and mortality rates rising at an alarming pace worldwide. Relevant projections suggest that by 2030, CVDs will be responsible for around 23.6 million deaths every year across the world [1]. Atherosclerosis, a chronic inflammatory vascular disorder, is widely acknowledged as the primary underlying cause of nearly all CVDs [2]. The pathological lesions of atherosclerosis develop over a long period of time, marked by the gradual accumulation and transformation of lipids, inflammatory immune cells, vascular smooth muscle cells, and necrotic debris, which eventually form atherosclerotic plaques in the intimal region beneath the endothelial cell monolayer that lines the inner walls of blood vessels [3]. As these plaques grow over time, they can narrow the arterial lumen, impede blood flow, and trigger tissue ischemia. Worse still, unstable plaques are prone to rupture; if this happens in the coronary arteries, it can induce local thrombosis that completely blocks blood flow, leading to acute myocardial infarction. In other cases, the thrombus may break off from the coronary site, travel to the cerebral vessels, and result in an ischemic stroke [4,5]. In recent years, the rising incidence of atherosclerosis has not only posed a severe threat to public health but also placed a heavy economic burden on global healthcare systems.

Currently, most conventional clinical interventions for atherosclerosis focus on normalizing blood lipid profiles, yet these strategies often lack direct inhibitory effects on the progression of existing atherosclerotic plaques [6,7]. For this reason, there is an urgent need to explore bioactive substances that can directly interfere with the development of atherosclerosis. In this context, natural active compounds derived from edible plants have drawn particular research interest, as these food-derived components are generally well-tolerated by the human body and can be safely used for long-term daily intake [8]. These natural food bioactives exhibit unique advantages in atherosclerosis prevention: they can inhibit oxidative stress, regulate the release of vasoactive substances, reduce systemic inflammation, and balance the coagulation–anticoagulation system, acting through multiple pathways, links and targets to exert comprehensive regulatory effects [9,10].

Octacosanol is a natural long-chain fatty alcohol, and it is the core functional nutrient component of plant wax esters [11]. Existing studies have shown that octacosanol can regulate motor function in both humans and animal models, and it also plays a modulatory role in energy metabolism, blood coagulation, nervous system function, inflammatory responses, and oxidative stress status [12,13]. These findings point to the great potential of octacosanol in the dietary prevention of atherosclerosis. However, current research on this topic is still relatively limited, and its specific efficacy and the underlying molecular mechanisms require more in-depth exploration. A previous review by Zhou et al. [14] has provided a broad overview of the wide-ranging health benefits of octacosanol, covering diverse bioactivities including anti-fatigue, anti-hypoxia, neuroprotective, immunomodulatory and metabolic regulatory effects. Nevertheless, no dedicated review has systematically focused on the anti-atherosclerotic efficacy and underlying cardiovascular protective mechanisms of octacosanol to date. Distinct from existing general reviews on octacosanol, this work exclusively targets the pathological progression of atherosclerosis and systematically elaborates the multi-target protective effects of octacosanol across core pathological links as well as the latest advances in nano-delivery strategies and clinical combination therapy. Therefore, conducting a comprehensive review and analysis of octacosanol’s anti-atherosclerotic effects and its working mechanisms is of great importance. As a narrative review summarizing available research evidence, this work will not only help us fully understand the biological functions of this natural active compound, but also maximize the health value of this natural resource, providing solid support for the advancement of atherosclerosis dietary prevention. Furthermore, it will lay a theoretical foundation for the development of octacosanol as a natural dietary supplement and its application as a core ingredient in functional food products.

## 2. Octacosanol Sources and Properties

### 2.1. Sources of Octacosanol

Octacosanol mainly exists in the form of wax and is abundantly distributed in the epidermis of leaves, stems, fruits and other parts of many plants [15]. It presents as white powder or scaly white crystalline solid, odorless, non-hygroscopic, and shows good stability against light, heat, acids and alkalis [15]. When its purity exceeds 97%, it has a melting point of 83.2–83.6 °C and a relative density of 0.783 at 85 °C. Common extraction technologies for octacosanol include supercritical fluid extraction, organic solvent extraction, saponification separation, transesterification separation, molecular distillation and ultrasonic-assisted extraction [14]. Natural octacosanol is widely present in various natural sources, most commonly as the major bioactive component of policosanols—a class of heterogeneous mixtures of saturated long-chain aliphatic alcohols (C20–C34) isolated from plant waxes such as sugarcane wax, rice bran wax and beeswax. Octacosanol (C28) typically constitutes ~60% of policosanol mixtures and is widely recognized as the primary constituent responsible for the lipid-lowering and cardiovascular protective effects attributed to policosanols [16,17]. Other long-chain aliphatic alcohols in the policosanol family, including hexacosanol (C26) and triacontanol (C30), also exhibit mild lipid-modulating and anti-inflammatory activities, but their efficacy profiles and underlying mechanisms remain far less characterized compared with octacosanol. It has been reported that octacosanol can be extracted from the wax of tomato peels, grape peels, apple peels, sugarcane, beeswax, and rice bran wax [18,19,20]. Among these sources, rice bran is the most important raw material for octacosanol extraction at present, due to its abundant output and low cost. In addition, while the technology for extracting octacosanol from rice bran is currently the most advanced, yields remain relatively low [14]. Further optimizing the extraction process of octacosanol from rice bran can not only improve the economic added value of rice bran, but also promote the high-value utilization of grain and oil processing by-products, which has great potential for promoting the industrialization of octacosanol-related products.

**Figure 1:**
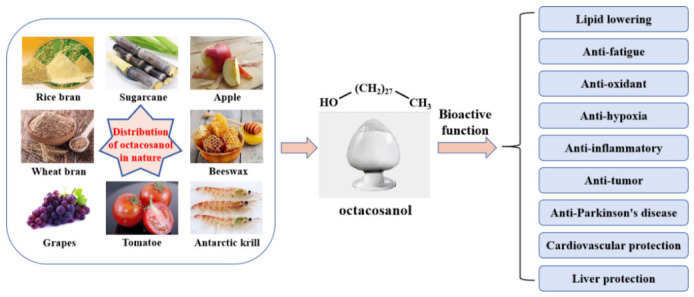
Sources and bioactive functions of octacosanol. Octacosanol is a natural long-chain fatty alcohol found in diverse sources such as rice bran, sugarcane and wheat bran, and exhibits a wide range of bioactive functions including lipid-lowering, antioxidant, anti-hypoxia, anti-inflammatory, and cardiovascular protection.

Studies have shown that octacosanol has extremely high biological safety. Oral toxicity studies in mice showed that its median lethal dose (LD50) is as high as 18,000 mg/kg, which is far higher than that of table salt (LD50 = 3000 mg/kg) [14]. Octacosanol has a variety of biological activities, including anti-fatigue, anti-hypoxia, antioxidant, anti-inflammatory and anti-tumor properties [21]. In addition, it can regulate immune function and energy metabolism, and has potential health benefits for cardiovascular diseases, cerebrovascular diseases, diabetes, Parkinson’s disease and other conditions [14]. The high biological safety of octacosanol makes it widely applicable in food, pharmaceuticals and cosmetics. It has long been a topic of intensive research in the development of functional foods and dietary supplements. At present, octacosanol has been widely added to various food products, such as functional beverages, candy, chocolate, biscuits, pastries, as well as dietary supplement products like capsules and tablets [22]. The sources and bioactive functions of octacosanol are illustrated in Figure 1 and Table 1.

**Table 1:** Overview of octacosanol’s physiological benefits and mechanisms.

Physiological Benefit	Mechanism of Action	Key Targets (Pathways)	References
Anti-Fatigue	Increases glycogen storage (LG, MG); reduces metabolic byproducts (BLA, LDH) and (CK, BUN); enhances antioxidant enzymes (SOD, GSH-Px); regulates fatigue-related genes (Trim63, Prx, Bcl3, Mybpc3)	SOD, GSH-Px, LDH, BLA, LG, MG; BUN, CK; Trim63, Prx, Pmp22, Ulk3, Arrdc2, Mybpc3, Bcl3, Cacna1h, Ca^2+^-ATPase (GO)	[11,12,22]
Anti-Inflammatory	Suppresses MAPK/NF-κB/AP-1 and TLR4/MyD88/NF-κB signaling cascades; downregulates pro-inflammatory cytokines and mediators; reduces monocyte-endothelial adhesion; reshapes gut microbiota and elevates short-chain fatty acids	p38, JNK, ERK1/2, NF-κB, AP-1; TLR4, MYD88, TIRAP, TRAF6, IRAK1; TNF-α, IL-1β, IL-6, iNOS; sPLA2.	[13,17,23,24]
Lipid lowering	Activates AMPK via peroxisomal metabolism; inhibits SREBP-mediated lipogenesis and pancreatic lipase; modulates PPAR pathways and promotes BAT thermogenesis; attenuates statin-induced PCSK9 elevation; lowers TC/TG/LDL-C and elevates HDL-C; reduces aortic calcification and blood pressure	AMPK, SIRT1, SREBP-1c/2, FASN, PPARα/δ/γ, pancreatic lipase, UCP-1, PCSK9, LDL-R, Wnt3a, BMP-2	[16,25,26,27,28,29,30,31,32,33,34,35]
Anti-oxidant	Restores endogenous antioxidant enzyme activities; scavenges ROS and inhibits lipid peroxidation; activates Nrf2/ARE antioxidant signaling; improves systemic oxidative stress status	GSH, SOD, CAT, LPO, MDA, ROS, Nrf2, GPx-1, HO-1	[13,21,34,36]
Hepatoprotective	Reduces serum transaminase levels; restores hepatic GSH content; alleviates hepatic lipid accumulation; regulates hepatic AMPK/SREBP-1c signaling	ALT, AST, MPO, XO, GSH, AMPK, SREBP-1c, FASN, PPARα, LDLR	[26,27,36]
Vascular Endothelial Protection	Inhibits TLR4/NF-κB signaling and adhesion molecule expression; reduces monocyte-endothelial adhesion; maintains endothelial junction integrity; suppresses cytoskeletal remodeling	TLR4, NF-κB, VCAM-1, ICAM-1, selectins, VE-cadherin, ZO-1, β-catenin	[17,27]
Antiplatelet & Antithrombotic	Balances PGI_2_/TXA_2_ equilibrium; inhibits agonist-induced platelet aggregation; reduces atherosclerotic intimal thickening; lowers circulating endothelin level	TXA_2_, PGI_2_, COX, platelet aggregation (AA/collagen/ADP), endothelin	[37,38,39]
Antitumor & Anti-angiogenic	Inhibits matrix metalloproteinase activity; blocks NF-κB nuclear translocation and DNA binding; downregulates VEGF gene expression	MMP-2, MMP-9, NF-κB, VEGF.	[14]

### 2.2. Octacosanol Solubility and Bioavailability

The oral bioavailability of food-derived bioactive compounds is defined as the fraction of an ingested compound that reaches the systemic circulation in its biologically active form. This mainly depends on their chemical composition and molecular structure. For a functional ingredient to exert its health effects effectively, it must not only reach the target tissues and organs, but also achieve sufficient distribution to produce beneficial health effects. However, there are many obstacles that hinder the entry of bioactive compounds into the systemic circulation, such as chemical instability during the digestion process, insufficient solubility in body fluids, delayed gastrointestinal absorption, and first-pass metabolism. As a hydrophobic bioactive compound, octacosanol has limited water solubility due to its lipophilic properties and large molecular structure, which leads to insufficient absorption and low bioavailability, thus limiting its actual health benefits [40]. In a related study, researchers administered 100 mg/kg of policosanol (containing 62% octacosanol) to SD rats orally and evaluated the in vivo pharmacokinetics of octacosanol by analyzing plasma samples through gas chromatography-mass spectrometry (GC-MS) within 0 to 180 min after administration [41]. The results showed that the plasma concentration of octacosanol reached the peak at 60 min, with the concentration ranging from 160 to 510 ng/mL [41]. Another study found that after oral administration of 40 mg/kg octacosanol, the peak plasma concentration in rats was only 14–16 ng/mL [42]. These results indicate that octacosanol has a relatively low oral bioavailability. A recent analysis further delineated four core factors restricting its oral bioavailability: limited bioaccessibility due to extreme hydrophobicity, selective tissue distribution preferentially in metabolically active organs, low intestinal absorption efficiency with most unabsorbed octacosanol excreted unchanged in feces, and rapid metabolic transformation via peroxisomal β-oxidation and gut microbial metabolism [43]. Addressing the limited aqueous solubility and low bioavailability of hydrophobic functional ingredients remains a key challenge in food science and nutrition research.

### 2.3. Nanotechnology to Enhance the Bioavailability of Octacosanol

Nanoencapsulation using food-grade hydrophilic carriers represents a well-validated strategy to improve the aqueous solubility and oral bioavailability of hydrophobic functional ingredients. As systematically summarized by [43], established delivery strategies for octacosanol cover nanocomplexes, microcapsules, micelles, nanoemulsions and nanocrystals, among which protein-based nanocomplexes and food-grade nanoemulsions are the most widely investigated systems for cardiovascular health applications. Nano-encapsulation can not only protect the active compounds, but also isolate them from adverse environmental conditions during food processing, storage and transportation, thus improving their stability and biological activity [44]. Specifically, octacosanol can improve its solubility by forming complexes with specific food-grade nanocarriers. For example, soy protein can form a spherical conformation with a hydrophilic outer layer and a hydrophobic inner core. This unique structure allows it to encapsulate octacosanol in its core, forming a stable complex [45,46]. After treatment with strong alkali (at pH 10.0, 11.0, and 12.0), the tertiary structure of soy protein isolate (SPI) is denatured, which leads to the exposure of more hydrophobic regions and amino acid residues such as tryptophan (Trp) and tyrosine (Tyr). This structural change further promotes the formation of nanoparticle complexes between octacosanol and soy protein. The combination of protein nanoparticles and octacosanol leads to significant changes in particle size and morphology. The prepared nanocomplexes show high thermal stability and salt ion stability, and most of them can be uniformly dispersed in the aqueous phase [47]. To further improve the encapsulation efficiency of octacosanol, researchers have combined soy protein/octacosanol complexes with polysaccharides to form core-shell nanocomplexes. In this structure, octacosanol is encapsulated in the hydrophobic cavity of soy protein, and the additional polysaccharide layer plays a shielding role, which enhances the compactness of the encapsulation. As a result, the encapsulation efficiency of octacosanol is significantly improved [48].

Nanoemulsion is a homogeneous dispersion system formed spontaneously by the combination of water, oil, surfactants and cosurfactants. With droplet sizes in the nanoscale range, usually between 20 and 500 nm, nanoemulsions have unique properties [49]. These include large specific surface area, improved dissolution rate and solubility, as well as enhanced mucosal permeability. These properties make them promising carriers for the encapsulation, protection and delivery of lipophilic substances in the food, beverage and cosmetics industries [49,50]. First of all, nanoemulsions can improve the solubility of lipophilic substances by promoting the interaction between surfactants, cosurfactants and the functional groups of the active ingredients [51]. The higher the structural similarity between these molecules, the more significant the dissolution effect. Secondly, nanoemulsions have a large interfacial area and low surface tension, which allows them to pass through the hydration layer of the gastrointestinal wall in the form of small lipid droplets or by forming mixed micellar phases [52,53]. This promotes the absorption of lipophilic substances by small intestinal epithelial cells. Nanoemulsions are not only simple to prepare but also have strong practical applicability. They provide a promising way to improve the oral absorption of poorly water-soluble functional ingredients and enhance their bioavailability [54]. In our previous research, we successfully prepared oil-in-water nanoemulsions using the phase inversion method [55]. Specifically, we used PEG40-hydrogenated castor oil as the surfactant and ethyl acetate as the cosurfactant. This method significantly improved the solubility and bioavailability of octacosanol. The prepared nanoemulsion has excellent biological safety, and there is no obvious cytotoxic effect on colon cells (Caco-2) within the concentration range of 10–150 μg/mL. Notably, in an in vitro intestinal model constructed with continuously differentiated Caco-2 cell monolayers, the transmembrane transport efficiency of octacosanol nanoemulsion was 5.4 times higher than that of conventional octacosanol. In vivo experiments further showed that the oral intestinal absorption rate in rats was increased by about 2.9 times. In a follow-up study, researchers successfully prepared octacosanol nanoemulsions using octacosanol, olive oil, Tween 80, glycerin and water, with the weight percentages of these components being 0.1%, 1.67%, 23.75%, 7.92% and 66.65% respectively [11]. The prepared nanoemulsion had an average particle size of 12.26 ± 0.76 nm. It is worth noting that the nanoemulsion showed excellent stability under different pH conditions, as well as under cold, heat, ionic stress and long-term storage conditions. In addition, it also significantly improved the anti-fatigue activity of octacosanol. Although nanoemulsions have been widely studied, there are still some challenges. For example, the solubility of active ingredients in lipid components is insufficient, the loading capacity of functional ingredients is limited, and surfactants may cause gastrointestinal irritation [56,57]. Nanoemulsion technology is a promising method to improve the bioavailability of octacosanol, but there are still few relevant research reports on octacosanol nanoemulsions at present.

## 3. Pathogenesis of Atherosclerosis

Under normal physiological conditions, the human body maintains strict control over cholesterol metabolism and transport, including the fine balance between cholesterol influx into cells and its efflux out of cells. When this delicate regulatory balance is disrupted—whether due to increased cholesterol uptake or impaired efflux—it can set the initial stage for the development of atherosclerosis [58]. Low-density lipoprotein (LDL) and high-density lipoprotein (HDL) are two core lipoprotein particles in the blood circulation: LDL is responsible for transporting cholesterol from the liver to peripheral tissues, while HDL helps carry excess cholesterol from peripheral tissues back to the liver for excretion through the biliary system [59]. To date, it is a widely accepted consensus that elevated low-density lipoprotein cholesterol (LDL-C) levels are a key driver of atherosclerosis progression, whereas higher high-density lipoprotein cholesterol (HDL-C) levels offer a protective effect against this pathological process [60]. For this reason, intervention strategies for atherosclerosis prevention have long focused on lowering plasma LDL-C levels while boosting HDL-C levels.

Atherosclerosis is defined by the buildup of lipid deposits on the arterial wall, which eventually leads to plaque formation, and this process in turn triggers a strong immune response targeting these accumulated lipids [61]. The earliest lipid accumulations in the arterial intima, known as fatty streaks, are mainly composed of apolipoprotein B-containing lipoproteins, especially LDL remnants [62]. These trapped LDL particles are prone to oxidation, forming oxidized low-density lipoprotein (ox-LDL) [63]. The buildup of ox-LDL in the arterial intima then triggers an inflammatory response in the neighboring endothelial cells, prompting them to secrete pro-inflammatory cytokines and chemokines [63]. These inflammatory factors further alter the expression of key genes related to cholesterol metabolism and transport, such as APOE, ABCA1, ACAT1 and MSR1, which in turn promotes the formation of foam cells [60]. The aggregation of these foam cells marks the start of atherosclerotic lesion formation and drives the gradual progression of plaques.

**Figure 2:**
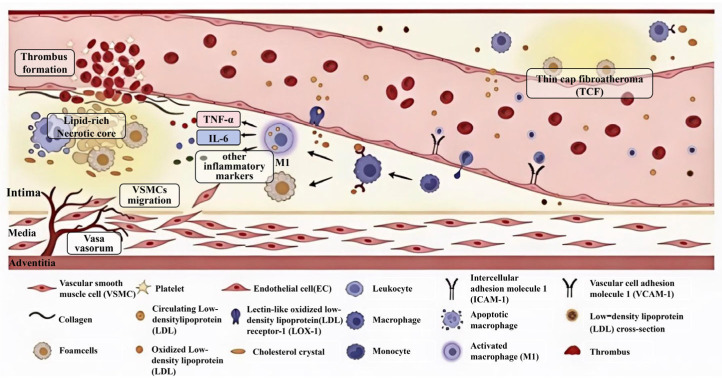
Schematic illustration of the progression of atherosclerosis [69]. (i) ox-LDL promotes the formation of foam cells; (ii) Inflammatory mediators (e.g., TNF-α, IL-6) and immune cells, including macrophages and monocytes, actively participate in the inflammatory response; (iii) Vascular smooth muscle cells contribute to the development of fibrous caps, while matrix degradation and cellular apoptosis lead to the formation of a lipid-rich necrotic core; (iv) Plaque rupture can subsequently trigger thrombosis, resulting in the obstruction of blood flow.

During the progression of atherosclerotic lesions, accumulated foamy cells undergo apoptosis and necrosis, releasing their lipid contents into the arterial intima (Figure 2). These apoptotic cells and lipids accumulate to form a lipid-rich necrotic core. In advanced stages of plaque development, macrophages, endothelial cells, and T cells contribute by stimulating the proliferation and migration of vascular smooth muscle cells from the media to the intima. The migrating vascular smooth muscle cells subsequently form a fibrous cap over the lipid core [64]. Additionally, these cells synthesize extracellular matrix (ECM) components, which reinforce the structural integrity of the fibrous cap [65]. Since the fibrous cap plays a critical role in stabilizing atherosclerotic lesions, the equilibrium between extracellular matrix (ECM) deposition and degradation is of paramount importance. This balance significantly influences the clinical progression of atherosclerosis. Activated macrophages and foamy cells secrete matrix metalloproteinases (MMPs), which facilitate ECM degradation and thereby increase the vulnerability of atherosclerotic plaques to rupture [66]. Following plaque rupture, platelets rapidly aggregate, leading to arterial occlusion or obstruction, which can culminate in coronary heart disease events.

Statins are the most widely used cholesterol-lowering drugs in clinical practice. They work by selectively inhibiting 3-hydroxy-3-methylglutaryl-CoA reductase (HMGCR), a key rate-limiting enzyme in hepatic cholesterol synthesis [67]. This inhibition reduces the body’s endogenous cholesterol production, and at the same time, statins upregulate the expression of LDL receptors (LDLr) on the surface of hepatocytes, which enhances the liver’s ability to take up and catabolize LDL particles. As a result, circulating LDL-C levels are significantly reduced. However, even with statin treatment, many patients still face a notable residual risk of cardiovascular events, and some patients even show obvious statin resistance [68]. What’s more, high-dose statin therapy is often associated with adverse reactions such as myalgia and hepatic dysfunction [68]. For these reasons, there is an urgent need for new intervention strategies that can be used either alone or in combination with statins. Right now, one of the most promising research directions for atherosclerosis prevention is the exploration of natural food-derived bioactive compounds, which can exert comprehensive regulatory effects through multiple pathways and targets.

## 4. Physiological Activity and Potential of Octacosanol Against Atherosclerosis

### 4.1. Antioxidant and Anti-Atherosclerosis

To clarify the formulation differences of test substances across cited studies and distinguish pure octacosanol from policosanol mixtures and commercial nutraceutical products, we have summarized the detailed information of relevant investigations in Table 2. Notably, two recent studies using highly purified octacosanol (>90–99%) have independently verified its intrinsic bioactivity independent of other policosanol components. Using primary human aortic endothelial cells, Tang et al. [17] demonstrated that purified octacosanol directly suppresses LPS-induced inflammatory responses via inhibiting the TLR4/MyD88/NF-κB cascade, reduces monocyte adhesion by downregulating adhesion molecules (VCAM-1, ICAM-1, P/E-selectins), and preserves endothelial barrier integrity by maintaining adherens and tight junction proteins, providing direct cellular-level evidence for octacosanol as the core anti-atherosclerotic constituent of policosanols. Koh et al. [16] further confirmed that non-esterified octacosanol alone modulates adipose lipid catabolism and thermogenesis through PPARα/δ and AMPK signaling pathways, and that fatty acid esterification can further enhance its bioavailability and metabolic efficacy. Under exposure to various noxious stimuli, the homeostatic balance between oxidant production and antioxidant defense is disrupted, triggering an oxidative stress response. Excessive production of reactive oxygen species (ROS) can induce cell damage, apoptosis, and ultimately result in cellular dysfunction. Increased ROS flux can react with proteins, lipids, and DNA, disrupting redox balance, causing lipid peroxidation in biological membranes, and further damaging endothelial cells [70]. Endothelial cells release nitric oxide (NO), which inhibits cell proliferation, reduces collagen fiber synthesis, and suppresses platelet activation and aggregation, thereby preventing the onset and progression of atherosclerosis [71]. With the increase in ROS, toxic peroxynitrite is generated, leading to reduced NO availability and subsequent uncoupling of endothelial nitric-oxide synthase (eNOS) [71]. Under normal physiological conditions, NO produced by eNOS scavenges superoxide anions, thereby protecting cells from oxidative stress-induced apoptosis.

Accumulating evidence suggests that octacosanol may inhibit ROS production via multiple signaling pathways, scavenge excessive ROS, and prevent the oxidative denaturation of NO. Additionally, it may mitigate uncoupling reactions, enhance eNOS expression, and maintain NO levels, thus combating oxidative stress [14]. Octacosanol exhibits potent anti-glycation and anti-apoptotic properties, as well as tissue regenerative activity. It has been shown to elevate serum HDL levels, thereby contributing to anti-aging effects and potentially extending lifespan [72]. Under conditions of oxidative stress, phospholipids and cholesterol esters enriched with polyunsaturated fatty acids (PUFAs) in cell membranes and lipoproteins are susceptible to oxidation. This oxidation proceeds via free radical-induced lipid peroxidation (LPO), resulting in a complex array of oxidation products. Extensive evidence indicates that these oxidized lipids play an active role in the inflammatory processes associated with atherosclerosis by interacting with immune cells, such as macrophages, and endothelial cells [73]. Our previous studies have demonstrated that in the lipid peroxidation reaction of the linoleic acid system, octacosanol nanoemulsions at concentrations ranging from 200–500 μg/mL exhibit significant inhibition of lipid peroxidation [55]. In a rat model of acute liver injury induced by carbon tetrachloride, supplementation with octacosanol mitigates the elevation in serum transaminase activity caused by carbon tetrachloride exposure [36]. Octacosanol can mitigate the elevation of myeloperoxidase, xanthine oxidase activities, and LPO levels in the liver. It also alleviates the reduction in superoxide dismutase (SOD) and lytic enzyme activities, as well as the decrease in glutathione content within the liver. In normal rats not exposed to carbon tetrachloride, octacosanol reduces hepatic LPO levels and increases glutathione content [36]. Furthermore, in mouse models, octacosanol effectively alleviates oxidative stress-induced damage, thereby mitigating the adverse effects of stress on sleep [74]. Collectively, although accumulated evidence supports the ROS-scavenging capacity of octacosanol, direct mechanistic evidence in the context of atherosclerosis remains limited. Further investigations using dedicated oxidative stress injury models are warranted to delineate its antioxidant mechanisms at the molecular level.

**Table 2:** Summary of octacosanol and policosanol formulations in cited studies.

First Author (Year)	Test Material	Purity (Content)	Study Model	Dosage (Concentration)	Reference
Zhu H (2024)	Octacosanol nanoemulsion (olive oil + Tween 80 + glycerol)	90%	Mice, exercise-induced fatigue	10, 30 mg/kg/day, intragastric, 30 d	[11]
Zhou Y (2021)	Pure octacosanol	99%	C57BL/6 mice, overexercise fatigue	200 mg/kg/day, intragastric, 30 d	[12]
Ding YY (2023)	Octacosanol	90%	C57BL/6 mice, HFD obesity/IR	10, 20, 30 mg/kg/day, intragastric, 10 wk	[13]
Koh YC (2025)	Nonesterified octacosanol, Lauric-acid-esterified octacosanol, Oleic-acid-esterified octacosanol	>90% (95% C28 + 5% C30)	Male C57BL/6J mice, HFD	150 mg/kg/day, diet mix, 11weeks	[16]
Tang J (2026)	Octacosanol	>99%	Primary HAECs, LPS inflammation	OCT 1.25, 2.5, 5 μM; LPS 100 ng/mL	[17]
He WS (2024)	Octacosanol lipoate	Raw > 95%, product > 99%	Sunflower oil, high-temp oxidation	200 ppm (equimolar to BHT)	[21]
Wang M (2024)	Octacosanol nanoemulsion (corn oil + Tween 80)	90%	Mice, anti-fatigue	100 mg/kg/day, intragastric, 30 d	[22]
Li D (2019)	SPI/octacosanol nanocomplex	99%	In vitro characterization	SPI 4%, OCT 5.5 mg/mL	[47]
Li D (2020)	SPI-octacosanol-polysaccharide core-shell nanocomplex	99%	In vitro characterization	SPI 4%, polysaccharide 1%	[48]
Jia M (2024)	O/W nanoemulsion (PEG-40 hydrogenated castor oil + ethyl acetate)	90%	Caco-2 cells; SD rats	5 mg/mL; in vitro 100 μg/mL; in vivo 80 mg/kg	[55]
Fernández-Arche A (2009)	Long-chain fatty alcohols (pomace olive oil)	C28 15.3%	RAW264.7; rat neutrophils	25, 50, 100 μg/mL; sPLA_2_ 1–100 μg/mL	[23]
Montserrat-De La Paz S (2014)	Long-chain fatty alcohols (evening primrose oil)	C28 7.64%	Murine peritoneal macrophages (LPS)	25, 50, 100 μg/mL	[75]
Guo T (2017)	Octacosanol	99%	RAW264.7 (LPS); DSS colitis mice	In vitro 10–100 μg/mL; in vivo 100 mg/kg/day	[24]
Molina V (1999)	Policosanol	~60–66% C28	Gerbils, unilateral carotid ligation	100, 200 mg/kg intragastric (every 12/24 h, 48 h)	[76]
Arruzazabala MdL (1993)	Policosanol	~60–66% C28	Gerbils, bilateral carotid clamping/reperfusion	100, 200 mg/kg intragastric (immediately post-clamping)	[37]
Arruzazabala MdL (2000)	Policosanol	~60–66% C28	Rabbits, 0.5% cholesterol diet	25, 200 mg/kg/day, 60 d	[39]
Sharma R (2019)	Octacosanol and policosanol	Octacosanol: Not specified; Policosanol: C28 18%	Male C57BL/6 mice, HFD-induced obesity	60 mg/kg/day, oral gavage, 4 weeks	[26]
Bai J (2022)	Octacosanol	Not specified	Male C57BL/6J mice, HFD-induced obesity	100 mg/kg/day, oral gavage, 10 weeks	[27]
Dong X (2025)	Octacosanol	>90%	Pancreatic lipase; HepG2 cells	IC_50_ = 7.87 ± 0.72 μg/mL; HepG2 10–40 μg/mL	[28]
Lee EY (2016)	Policosanol	C28 60–70%	Zebrafish HCD; BV-2 cells	Diet 0.003% PCO (0.3 μg/fish/day), 9 wk; in vitro 9–46 μM	[77]
Singh DK (2006)	Policosanol	~60% C28	McA-RH7777 hepatoma cells	5–25 μg/mL, 3 h	[29]
Menéndez R (1997)	Policosanol	~60–66% C28	Rabbits, casein-induced hypercholesterolemia	50 mg/kg/day, intragastric, 30 d	[78]
Banerjee S (2011)	Policosanol	~66% C28,	McA-RH7777 cells; mice	In vitro 15 μg/mL, 3 h; in vivo 25–100 mg/kg	[30]
Kamchonemenukool S (2025)	Lauric-acid-esterified octacosanol, Oleic-acid-esterified octacosanol	95% (C28) for esterification; natural C28 ~60%	Male C57BL/6J mice, HFD	150 mg/kg/day, diet mix, 11 weeks	[31]
Elseweidy MM (2018)	Policosanol	Not specified	Rabbits, 0.5% cholesterol diet	5 mg/kg/day, intragastric, 12 weeks	[79]

Note: C28 refers to octacosanol, and C30 refers to tricosanol.

### 4.2. Anti-Inflammatory and Anti-Atherosclerosis

During the early phase of atherogenesis, vascular endothelial cells (VECs) sustain injury, which triggers the secretion of endothelial-derived growth factors and the upregulation of adhesion molecules including P-selectin, vascular cell adhesion molecule-1 (VCAM-1), and intercellular adhesion molecule-1 (ICAM-1) [80]. These substances increase vascular permeability, allowing a significant amount of lipids in the bloodstream to penetrate the vascular intima. Concurrently, these changes facilitate the adhesion of circulating lymphocytes and monocytes to vascular endothelial cells. Monocytes are further attracted by monocyte chemoattractant protein-1 (MCP-1) and migrate through the endothelium into the intimal space [56]. Upon entering this region, monocytes are stimulated by cytokines to differentiate into macrophages. Macrophages then phagocytose lipids via scavenger receptors, leading to the formation of foamy cells. Studies have demonstrated that the pro-inflammatory cytokine tumor necrosis factor-α (TNF-α), produced by T cells and macrophages, plays a crucial role in the upregulation of adhesion molecules, chemokines and scavenger receptors [81]. TNF-α, along with interleukin-6 (IL-6), induces vascular endothelial cells to generate reactive oxygen species (ROS), which subsequently activates the nuclear factor kappa-B (NF-κB) signaling pathway. Activation of this pathway leads to increased expression of multiple inflammatory mediators, thereby exacerbating the inflammatory response and promoting cell proliferation [80]. This creates a self-perpetuating cycle that contributes to the progression and destabilization of atherosclerotic plaques. In the advanced stages of atherosclerosis, when plaque rupture and thrombosis occur, an inflammatory response plays a critical role. Activated macrophages secrete a wide array of matrix metalloproteinases (MMPs), which not only degrade interstitial collagen but also other components of the extracellular matrix, ultimately leading to plaque destabilization [66]. Additionally, inflammatory factors within the plaque induce the expression of platelet-derived growth factor (PDGF), exacerbating the migration and proliferation of vascular smooth muscle cells (VSMCs) and promoting microthrombus formation within the plaque [66].

**Figure 3:**
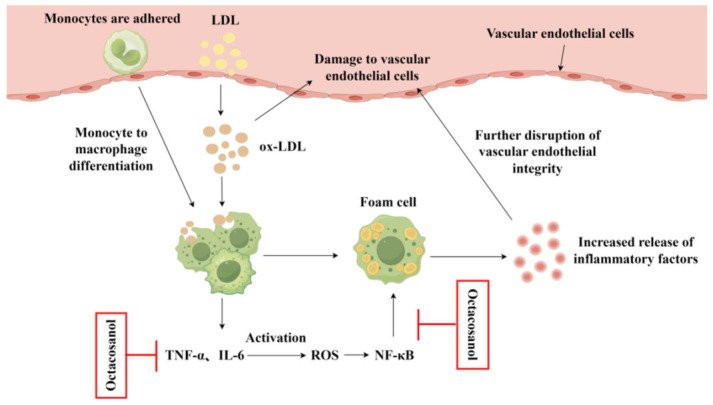
Anti-inflammatory mechanism of octacosanol. Octacosanol exerts inhibitory effects at multiple stages of atherosclerosis, including suppressing the secretion of pro-inflammatory cytokines such as TNF-α and IL-6 and preventing the formation of foam cells by inhibiting the ox-LDL-induced differentiation of monocytes into macrophages, thereby protecting vascular endothelial integrity and mitigating the inflammatory response.

The correlation between the anti-inflammatory activity of octacosanol and their hypolipidemic effects is illustrated in Figure 3. Octacosanol demonstrates potential anti-inflammatory effects by inhibiting TNF-α production. Within the concentration range of 25–100 μg/mL, octacosanol significantly reduces nitric oxide (NO) production in lipopolysaccharide (LPS)-stimulated macrophages in a dose-dependent manner [82]. Long-chain fatty alcohols, including octacosanol (15.3%) isolated from pomace olive oil, exhibit similar anti-inflammatory properties [23]. Additionally, these compounds reduce the production of TNF-α and prostaglandin E2, as well as significantly decrease thromboxane B2 (TXB2) levels in rat peritoneal neutrophils stimulated by the calcium ionophore A-23187 [23]. Octacosanol is associated with the inhibition of phospholipase A2 (PLA2) enzyme activity, achieving a 50% inhibition rate at a concentration of 6.2 μg/mL [23]. Consequently, octacosanol may exert regulatory effects on certain mediators involved in inflammatory processes. The long-chain fatty alcohols, including 7.64% octacosanol, extracted from Oenothera biennis L., can reduce the NO content in peritoneal macrophages of LPS-induced inflammatory mice [75]. These alcohols inhibit the expression of inducible nitric oxide synthase (iNOS) and the release of phospholipase A2 (PLA2) and thromboxane B2 (TXB2). Additionally, they suppress the expression levels of cyclooxygenase-2 (Cox-2), interleukin-1β (IL-1β), TNF-α and IL-6 [75]. Studies have demonstrated that octacosanol significantly blocks the expression of TNF-α, IL-6, IL-1β, and iNOS both in vitro and in vivo, and inhibits dextran sulfate sodium (DSS)-induced colitis in mice [24]. Furthermore, octacosanol downregulates the expression of phosphorylated p38 (p-p38) and phosphorylated c-Jun N-terminal kinase (p-JNK), thereby blocking the MAPK-NF-κB/AP-1 signaling pathway [24]. Octacosanol exerts anti-inflammatory effects through the regulation of gut microbiota. According to the study by Ding et al. [13], octacosanol significantly decreases plasma levels of Toll-like receptor 4 (TLR4), NF-κB, TNF-α and IL-6 in mice fed a high-fat diet (HFD) in a dose-dependent manner. This reduction mitigates HFD-induced oxidative stress and promotes the expression of the Nrf2/ARE signaling pathway. Octacosanol reshapes the gut microbiota by decreasing the relative abundance of Firmicutes at the phylum level while increasing that of Bacteroidetes and Verrucomicrobia [13]. These structural changes in the gut microbiota are significantly correlated with alterations in inflammatory biomarkers. Octacosanol may mitigate the release of inflammatory factors by inhibiting NF-κB activation in macrophages, thereby reducing foamy cell formation and protecting vascular endothelial cells. Future research should focus on conducting more in-depth studies to elucidate the specific targets of octacosanol in mitigating inflammation and fully leverage its advantages for the prevention and treatment of atherosclerosis.

### 4.3. Anti-Coagulation and Anti-Atherosclerosis

**Figure 4:**
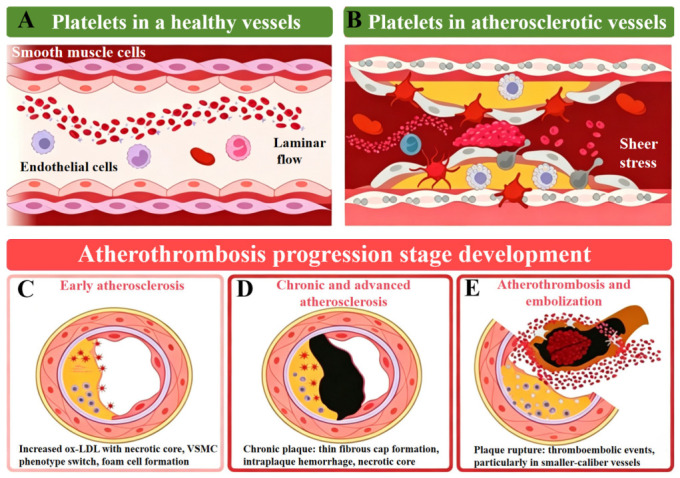
Platelet behavior and progression of atherothrombosis [84]. (**A**) Platelets in a laminar flow state in a healthy vessel; (**B**) Platelets subjected to shear stress in an atherosclerotic vessel, which is susceptible to life. The lower panels show the stages of atherothrombosis: (**C**) Early atherosclerosis as evidenced by increased oxidized LDL and foam cell formation; (**D**) Chronic/advanced atherosclerosis characterized by thinning of the fibrous cap and hemorrhage within the plaque; (**E**) Atherothrombosis/embolism formed after plaque rupture.

Upon endothelial injury, the extrinsic coagulation cascade is activated, which in turn drives platelet activation and the sequential activation of coagulation factors. A large amount of thrombin is then produced, which converts fibrinogen into fibrin, eventually leading to the formation of thrombus [83]. The integrity of vascular endothelium is the foundation of anticoagulation. The endothelium can synthesize and secrete a variety of anticoagulant and antithrombotic substances, which maintain the balance of the coagulation system. The hypercoagulable state of blood, as well as enhanced platelet aggregation and adhesion, plays a key role in the progression of atherosclerosis and the occurrence of its complications [84]. After the rupture of atherosclerotic plaques, the local release of procoagulant factors such as tissue factor is increased. This mediates the increase of platelet aggregation and adhesion, thereby promoting thrombosis [84]. As a result, arterial occlusion occurs, increasing the risk of acute atherosclerotic events (Figure 4).

Octacosanol can inhibit platelet activation through multiple pathways. It can not only inhibit platelet adhesion and aggregation, but also reduce the activity of various coagulation factors, thereby maintaining the balance of the coagulation system and preventing thrombosis. Specifically, octacosanol may exert an anticoagulant effect by inhibiting the production of thromboxane A2 (TxA2), which is a strong vasoconstrictor and platelet aggregator released by activated platelets [37,76]. Policosanol, which is mainly composed of octacosanol, has a significant effect in inhibiting platelet aggregation. In a study, Mongolian gerbils were given 200 mg/kg of policosanol for a long time [25]. The results showed that the mortality of cerebral infarction in the treated gerbils was significantly reduced. This effect is likely due to the ability of octacosanol to reduce the concentrations of arachidonic acid, collagen and adenosine diphosphate (ADP), thereby preventing their synergistic induction of platelet aggregation. GastnoG et al. [38] conducted a study on elderly diabetic patients, giving them octacosanol at a dose of 10 mg/day for 42 days. The results showed that octacosanol significantly inhibited platelet aggregation induced by arachidonic acid (the inhibition rates were 45% and 70% at concentrations of 1.5 and 3 mmol/L respectively), ADP (70% inhibition rate at 1 mmol/L) and collagen (20% and 17% inhibition rates at 0.5 mmol/L respectively). These results indicate that octacosanol has a strong inhibitory effect on platelet aggregation. Arruzazabal et al. [39] further studied the concentration-dependent effect of policosanol on the inhibition of platelet aggregation. The results showed that within the concentration range of 5–20 mg, the inhibition rate of platelet aggregation increased proportionally with the increase of policosanol concentration. At present, the research on the anti-platelet aggregation properties of octacosanol is still not sufficient, and its specific effects and mechanisms need to be further studied and verified.

### 4.4. Lipid-Lowering and Anti-Atherosclerosis

Dyslipidemia and hypercholesterolemia are well-recognized causal risk factors for cardiovascular diseases, with a robust causal association with atherosclerosis development. Plasma lipids, also known as blood lipids, primarily consist of cholesterol, triglycerides, phospholipids, and free fatty acids. While these lipids are essential nutrients for the human body, excessive levels can lead to lipid metabolism disorders [85]. Elevated blood viscosity and lipid deposition on the vascular intima result in plaque formation over time [58]. Hyperglycemia, hypertriglyceridemia, hypercholesterolemia, and elevated LDL levels each independently contribute to an increased risk of atherosclerosis [62]. It has been reported that certain long-chain fatty alcohols, particularly those with 20 to 36 carbon atoms, have demonstrated efficacy in reducing TC and LDL-C levels. In a study, rats were orally administered octacosanol at a dose of 60 mg/kg for 4 weeks [26]. This treatment not only prevented obesity induced by an HFD but also led to reductions in body weight and liver lipid content. Furthermore, octacosanol inhibited the hypertrophy of adipocytes in brown adipose tissue (BAT).

In the human body, a significant portion of high cholesterol originates from daily dietary intake. Non-metabolized lipids tend to accumulate and deposit in the liver, leading to inflammation and lipid peroxidation damage. According to the study by Bai et al. [27], after administering octacosanol via intragastric injection at a dose of 100 mg/kg/day for 10 weeks, the body weight, as well as the weights of the liver and adipose tissue in HFD mice, were significantly reduced. The plasma levels of TC, TG and LDL-C also decreased. Beyond hepatic lipid metabolism regulation, octacosanol lowers circulating lipids by inhibiting intestinal fat absorption. A recent study isolated octacosanol from Moringa oleifera leaves and identified it as a reversible mixed-type pancreatic lipase inhibitor (IC_50_ = 7.87 ± 0.72 μg/mL); it retained 73.5% of lipase inhibitory activity after simulated gastrointestinal digestion and significantly reduced intracellular TC and TG levels in oleic acid-treated HepG2 cells, uncovering a new lipid-lowering target of octacosanol [28]. H&E staining revealed that, compared with the hyperlipidemia group, supplementation with octacosanol reduced the size of fat droplets in liver tissue and adipocytes. A clinical trial involving 40 adolescents with hereditary hypercholesterolemia demonstrated that after consuming a diet containing 10 mg of octacosanol, TC levels decreased significantly by 18.5% (*p* < 0.001), LDL-C levels decreased significantly by 25.1% (*p* < 0.001), and apolipoprotein B (Apo-B) levels decreased significantly by 25.3% (*p* < 0.001) [25]. A study involving 120 male patients with hyperlipidemia found that, compared with those who received octacosanol or simvastatin alone, the patients who received both simvastatin and octacosanol concurrently exhibited a significantly higher reduction rate in LDL-C and TC levels [86]. In addition, octacosanol has been shown to increase HDL-C levels in hyperlipidemic zebrafish, reduce TC levels, and alleviate fatty liver symptoms. It also promotes the proliferation of microglial cells BV-2 and enhances tissue regeneration capacity. Furthermore, octacosanol inhibits cholesterol ester transfer protein (CETP) activity [77]. Recent studies have demonstrated that Armolipid Plus, a nutritional supplement containing octacosanol, can lower blood pressure, reduce TC and LDL-C concentrations, and promote glucose metabolism. Additionally, it modulates the ratio of apolipoprotein B to apolipoprotein A-I without significant side effects [87]. To provide a critical, evidence-based overview of clinical research, we systematically summarized all relevant clinical trials on the cardiovascular effects of octacosanol and policosanol in Table 3.

Octacosanol has been shown to inhibit cholesterol synthesis in both animal models and cell cultures, suggesting its potential as a mechanism for reducing blood cholesterol levels. Studies have demonstrated that policosanol, of which octacosanol is the primary component, can effectively lower LDL-C [88]. This effect is attributed to policosanol’s ability to reduce the activity of HMG-CoA reductase, thereby inhibiting cholesterol synthesis in cultured cells [29] and animals [78]. This inhibitory effect is associated with the activation of adenosine monophosphate-activated protein kinase (AMPK) and the phosphorylation of HMG-CoA reductase [30]. Notably, a recent in vivo study demonstrated that fatty acid esterification markedly enhances the hypolipidemic efficacy of octacosanol. In HFD-fed C57BL/6J mice, oleic-acid-esterified octacosanol (OEO) and lauric-acid-esterified octacosanol (LEO) both alleviated obesity, reduced serum TC, TG and LDL-C, and elevated HDL-C, with OEO showing superior activity. Mechanistically, esterified octacosanol suppresses fatty acid synthesis via the SIRT1/AMPK/SREBP-1c axis and inhibits cholesterol synthesis by downregulating SREBP-2 and its downstream enzymes HMGCS1 and HMGCR, providing robust in vivo validation of octacosanol’s lipid-modulating mechanisms [31]. Notably, the activation of AMPK is sustained by the oxidation of long-chain fatty alcohols, which constitute policosanol, into fatty acids. Furthermore, in intervention studies, supplementation with inositol has been shown to increase HDL-C, thereby contributing to a reduction in blood pressure [32]. Interestingly, several studies have demonstrated that in patients initiating statin therapy, policosanol can prevent the elevation of proprotein convertase subtilisin/kexin type 9 (PCSK9) levels. Additionally, it has been observed to modestly reduce PCSK9 levels in healthy volunteers [33]. PCSK9 promotes the degradation of LDLr, thereby influencing the clearance of LDL-C from circulation [89]. These findings suggest that octacosanol may serve as an effective adjuvant therapy for hyperlipidemia. However, further research is necessary to elucidate its precise mechanism of action.

**Table 3:** Summary of clinical trials investigating octacosanol for cardiovascular health management.

First Author	Population	Sample Size	Design	Intervention	Dosage	Duration	Primary Outcomes	Side Effects	Reference
Guardamagna O (2011)	Children 8–16 y, FH/FCH	40 (38 completed)	Double-blind, RCT, cross-over	Red yeast rice 200 mg + policosanol 10 mg	1 tablet/day	8 week	TC ↓18.5%, LDL-C ↓25.1%, ApoB ↓25.3% (all *p* < 0.001); TG ↓; HDL-C and ApoA-I unchanged	No serious AEs; 2 mild CPK elevations resolved	[25]
Castaño G (1999)	Older type II hypercholesterolemia	Not specified	Randomized, double-blind	Policosanol vs. Pravastatin	10 mg/day	8 week	Policosanol: LDL-C ↓19.3%, TC ↓13.9%, HDL-C ↑18.4% (*p* < 0.001), TG ↓14.1% (*p* < 0.01); platelet aggregation inhibition superior to pravastatin	2 pravastatin patients withdrew	[38]
Tang M (2013)	Male hyperlipidemia	Not specified	Randomized, controlled	Policosanol + simvastatin	Not specified	Not specified	Abstract only; specific data limited	Not reported	[86]
Lee EY (2016)	Healthy Korean subjects (young/middle-aged)	YN *n* = 7, YS *n* = 7, MN *n* = 11	Randomized, double-blind, placebo-controlled	Cuban policosanol	10 mg/day	8 week	SBP ↓4% (7 mmHg, *p* = 0.022); TG: YN ↓28%, MN ↓26%; HDL-C/TC: YN ↑36%, YS ↑35%, MN ↑8%; CETP activity ↓21–32%; glucose and uric acid ↓; LDL oxidation markedly ↓	No AEs reported	[77]
Marazzi G (2015)	Statin-intolerant CHD patients	100 (nutraceutical 50, ezetimibe 50)	Single-blind, randomized	Armolipid Plus^®^ (policosanol 10 mg)	1 tablet/day	3–12 mo	3 mo: LDL-C ↓26.8%, TC ↓18.8%, TG ↓13.2%, HDL-C ↑8.3%; 14 (28%) reached target; 12 mo: 58 (73%) in combination group reached target	No AEs; no transaminase/CK elevations	[87]
Park HJ (2019)	Healthy Korean, prehypertension	84 randomized, 76 completed	Double-blind, RCT, placebo-controlled	Cuban policosanol	10 or 20 mg/day	12 week	20 mg: peripheral SBP ↓7.7%, DBP ↓7.1%, aortic SBP ↓8.3%, TC ↓8.6%, LDL-C ↓18%, %HDL-C ↑5.3 pp; 10 mg: TC ↓9.6%, LDL-C ↓21%, %HDL-C ↑5.7 pp	No serious AEs	[32]
Guo YL (2014)	Atherosclerosis patients/healthy volunteers	Protocol I:26; II:15	Randomized, open-label	Atorvastatin ± policosanol	Policosanol 20 mg/day	8/12 week	Atorvastatin alone: PCSK9 ↑39.4% (*p* = 0.002); combination: PCSK9 ↑17.4% (*p* = 0.184); policosanol alone: trend toward PCSK9 ↓ (*p* = 0.069)	Well tolerated	[33]
Ciric MZ (2021)	Chronic statin therapy patients	87 (final 81)	Double-blind, RCT, placebo-controlled	Octacosanol 20 mg + VK2 45 μg	1 capsule/day	13 week	PCSK9-LDL-C positive correlation restored (supplement: r = 0.409, *p* = 0.012; placebo: r = −0.103, *p* = 0.508); absolute PCSK9 levels unchanged	AST/ALT improved	[35]

### 4.5. Octacosanol as a Supplement to Cardiovascular Disease Medications

The cholesterol in human plasma originates from two sources: dietary intake and de novo synthesis by cells. Inhibitors of 3-hydroxy-3-methylglutaryl coenzyme A reductase (HMGCR), collectively termed statins, are first-line and highly efficacious therapeutic agents for dyslipidemia [67]. Statins competitively bind to the catalytic site of HMGCR, thereby blocking the conversion of HMG-CoA to mevalonate, which is a rate-limiting step in cholesterol biosynthesis [90]. By inhibiting de novo cholesterol synthesis, statins upregulate the expression of LDLr, leading to reduced serum cholesterol levels and consequently lowering cardiovascular risk [91]. Modulation of the redox system by statins represents another crucial mechanism underlying their beneficial effects on the cardiovascular system [68]. Statins may exert a protective effect against oxidative stress (OS) in atherosclerotic tissues. However, they can also induce hepatotoxicity, nephrotoxicity, and myotoxicity via OS [92]. The combined use of statins with antioxidants derived from natural plants may produce a synergistic effect, thereby reducing statin-associated adverse reactions [91]. This approach is particularly beneficial for patients who cannot tolerate high-dose statin therapy.

Supplementing octacosanol to statin treatment can further improve the lipid profile and redox status markers in the responsive group, thereby promoting beneficial cardioprotection [34]. A recent study evaluated several standard lipid status biomarkers and assessed the responses of redox status markers and antioxidant defenses to both standard lipid-lowering therapy (atorvastatin) and combination therapy (atorvastatin + octacosanol) [34]. The 13-week supplementation with octacosanol significantly reduced LDL-C levels in subjects (*p* < 0.001), while simultaneously enhancing the antioxidant capacity of all participants. This effect may be partly attributed to the pleiotropic effects of statins and octacosanol, as well as a compensatory mechanism related to increased mitochondrial ROS production and apoptosis.

**Figure 5:**
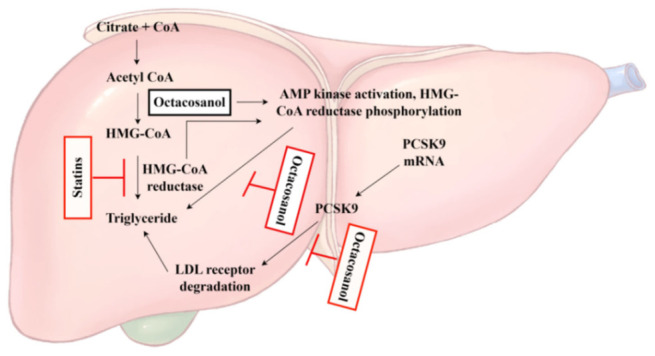
Lipid-lowering mechanism of octacosanol. Octacosanol exerts multiple lipid-modulating effects: it activates AMPK to promote the phosphorylation of HMG-CoA reductase, inhibits LDL receptor degradation, thereby enhancing clearance of blood LDL, and reduces both PCSK9 mRNA expression and protein production.

PCSK9 and PCSK9 inhibitors have garnered significant attention over the past decade. PCSK9, a protease, facilitates the lysosomal degradation of LDLr, thereby regulating circulating LDL-C levels [93]. This mechanism has spurred the development of a new class of drugs known as PCSK9 inhibitors, which effectively block the interaction between PCSK9 and LDLr, reducing LDL-C levels by up to 70% [94]. Studies investigating the interplay between statins and PCSK9 reveal that statin therapy increases serum PCSK9 levels, potentially diminishing the overall lipid-lowering efficacy of statins [95,96]. The existing HMG-CoA reductase inhibitors (statins) and PCSK9 inhibitors demonstrate significant potential in achieving LDL-C treatment goals [97]. However, lifelong statin prescriptions can lead to various side effects. Consequently, nutraceuticals with dual inhibitory activities against HMGCR and PCSK9 have garnered considerable attention. Research has shown that octacosanol effectively reduces HMG-CoA reductase activity and inhibits PCSK9 expression in both animal models and healthy human populations [29,97]. Ciric et al. [35] evaluated the potential of a dietary supplement (DS) containing octacosanol (20 mg) and vitamin K2 (45 mcg) to restore the disrupted physiological relationship between LDL cholesterol and serum PCSK9 in 42 patients who had been on long-term atorvastatin therapy. After 13 consecutive weeks of treatment, it was demonstrated that octacosanol could re-establish the physiological correlation between PCSK9 and LDL-C that had been disrupted by statins (r = 0.409, *p* = 0.012). These findings suggest that dietary supplements may be beneficial for potential responders, while caution should be exercised in recommending them for non-responders. The dual inhibitory mechanism of octacosanol on HMGCR and PCSK9 is illustrated in Figure 5. Future research should explore the effects of octacosanol supplementation at different time points, over longer periods, and/or with varying dosing regimens.

## 5. Conclusions and Prospects

Atherosclerosis is a chronic inflammatory disease, and its pathogenesis involves multiple pathological processes such as lipid metabolism disorders, oxidative stress, endothelial dysfunction, inflammatory response and coagulation system imbalance. Current clinical management of atherosclerosis primarily targets lipid profile normalization, but this lipid-lowering-only strategy is insufficient to halt atherosclerotic progression at its fundamental pathological origins. For example, although the current mainstream statins can effectively reduce LDL-C levels, their effect on plaque stability is relatively limited, and they are also accompanied by adverse reactions such as muscle toxicity and liver damage [68]. Therefore, exploring natural active compounds that can achieve multi-target regulation has become an important direction in the field of atherosclerosis prevention.

As a naturally occurring long-chain aliphatic alcohol, octacosanol has demonstrated substantial potential for the dietary prevention of atherosclerosis. Octacosanol can synergistically intervene in the pathological process of atherosclerosis through multiple mechanisms. Specifically, preclinical evidence supports that octacosanol may improve vascular endothelial function by scavenging reactive oxygen species (ROS), inhibiting lipid peroxidation (LPO), and restoring endothelial nitric oxide synthase (eNOS) activity [73]. In addition, it inhibits the release of pro-inflammatory factors such as TNF-α, IL-6 and IL-1β by inhibiting signaling pathways such as NF-κB and MAPK, while downregulating the expression of iNOS and COX-2 to block macrophage polarization and foam cell formation [80]. By inhibiting HMG-CoA reductase activity and reducing PCSK9 expression, octacosanol reduces cholesterol synthesis and enhances LDL-C clearance. In addition, the activation of the AMPK pathway promotes fatty acid oxidation, thereby alleviating high-fat diet-induced obesity and liver lipid deposition [30,78]. Moreover, by inhibiting the production of thromboxane A2 (TXA2) and platelet aggregation, it reduces the hypercoagulable state of blood, effectively reducing the risk of thrombosis after plaque rupture [37,76]. Together, these multi-target effects form a comprehensive regulatory network of octacosanol against atherosclerosis.

The core advantages of octacosanol in anti-atherosclerosis are as follows: (1) Multi-target regulation: it simultaneously interferes with key links of lipid metabolism, inflammation and thrombosis in atherosclerosis, thus complementing the role of statins; (2) High safety: animal experiments have shown that its LD50 is as high as 18,000 mg/kg, far higher than conventional drugs, making it suitable for long-term daily intake; (3) Synergistic effect: when used in combination with statins, it can enhance lipid-lowering efficacy while reducing side effects, providing a new idea of “natural-synthetic” combined health management.

Although the anti-atherosclerosis effects of octacosanol have been initially confirmed, low bioavailability, insufficient in-depth mechanism research, and lack of clinical evidence are still the main factors restricting its future application. (1) Bioavailability bottleneck: The high hydrophobicity of octacosanol leads to poor oral absorption (plasma concentration can only reach ng/mL level), which limits its in vivo efficacy [54]. Although nanoemulsion technology can partially improve solubility, challenges such as low drug loading, instability and potential gastrointestinal toxicity still hinder its industrial application [56,57]. (2) Fragmentation of mechanism research: Current research mainly focuses on individual pathways (such as NF-κB or AMPK), ignoring the comprehensive research on multi-target synergistic effects and cell-specific mechanisms, especially the key cells in atherosclerosis (such as endothelial cells, macrophages, smooth muscle cells). (3) Insufficient clinical evidence: Most of the current studies are based on animal or cell models, the clinical data is limited, the sample size is small, and there is a lack of long-term safety assessment and comparative analysis with other lipid-lowering substances [29,79]. (4) Standardization and quality control: The purity of octacosanol from different sources (such as rice bran wax, beeswax) is different, which may affect its efficacy. Therefore, it is necessary to establish a standardized system for purity and activity evaluation.

Future research can achieve breakthroughs in multiple dimensions. First, it is necessary to develop innovative octacosanol delivery systems to improve its targeting and stability. Nano-delivery technologies such as liposomes, solid self-microemulsions, or exosomes can be used to solve the bioavailability bottleneck, thereby providing technical support for overcoming this challenge. Second, integrating cutting-edge technologies such as single-cell sequencing and spatial transcriptomics can deeply analyze the anti-atherosclerosis mechanism of octacosanol. This approach can systematically clarify the cell-specific regulatory network of octacosanol in endothelial damage, plaque formation and rupture and identify its direct targets. These findings will provide a theoretical basis for multi-target dietary intervention strategies. In conclusion, octacosanol has great potential as a promising functional food ingredient for cardiovascular health maintenance. However, its wide application still requires interdisciplinary collaboration and technological advancement.

## Data Availability

The authors confirm that the data supporting the findings of this study are available within the article. All data generated or analyzed in the current study are available from the corresponding author upon reasonable request.
